# Chikungunya Fever - Epidemic in Rural Maharashtra

**DOI:** 10.4103/0970-0218.58409

**Published:** 2009-10

**Authors:** Nadeem Ahmad

**Affiliations:** Department of Community Medicine, Rural Medical College, PIMS, Loni-Bk - 413 736, District Ahmednagar, Taluka Rahata, Maharashtra, India. E-mail: nadeemarman@rediffmail.com; dr_nadeem_gsvm@yahoo.co.in

Sir,

Chikungunya fever, an arboviral infection, transmitted by the *Aedes aegypti* mosquito is caused by the chikungunya virus of family Togaviridae and genus *Alphavirus*. The illness can be severe, but it is self-limiting and nonfatal.

We conducted a cross-sectional study in the epidemic-affected Shrirampur town. A team of medical doctors from Rural Medical College, Pravara Institute of Medical Sciences, Loni, District Ahmednagar, carried out a house-to-house survey. A population of 1550 was covered. As per our clinical criteria, all fever cases with joint pains or arthralgia were cases of chikungunya fever unless proved otherwise. The objective of the study was to study the magnitude of the outbreak and to identify the possible socio-environmental factors responsible for the chikungunya fever epidemic in Shrirampur town in district Ahmednagar of Western Maharashtra during April/May 2006.

Out of the total 1550 population surveyed in Shrirampur town, there were 918 (59.2%) clinically suspected chikungunya fever cases; of these 55.7% were male and 44.3% were female. The main symptoms were acute-onset fever and joint pains (100%). About 75% of the cases were in the age range of 11–50 years, that is the active age-group [Table 1]. The majority of the cases (30.2%) were poor, belonging to social class IV of the modified Prasad's classification; 26.0% were illiterate. Apart from fever with joint pains, which was present in all cases of chikungunya fever, the other symptoms were severe headache (82.1%), arthralgia (81.0%), nausea/vomiting (62.0%), abdominal pain (34%), and a rash (14.6%) that was usually confined to the lower limbs and trunk. There was no hemorrhagic rash, thus helping to exclude dengue hemorrhagic fever.

**Table 1:** Age- and sex-wise distribution of the chikungunya fever cases

Age-groups	Males No. (%)	Females No. (%)	Total No. (%)
1–10	56 (10.95)	38 (9.33)	94 (10.23)
11–20	129 (24.65)	84 (20.63)	213 (23.20)
21–30	97 (18.98)	96 (23.58)	193 (21.02)
31–40	79 (15.45)	88 (21.62)	167 (18.19)
41–50	75 (14.67)	49 (12.03)	124 (13.50)
51–60	44 (8.61)	29 (7.12)	73 (7.95)
> 60	31 (6.06)	23 (5.65)	54 (5.88)
Total	511 (55.66)	407 (44.34)	918 (100.00)

Blood samples were taken by venipuncture from a random sample selected from amongst the 918 clinically suspected chikungunya fever cases. Out of 50 blood samples taken, 48 (96.0%) were reported as positive for the chikungunya virus using the diagnostic test kits procured from the National Institute of Virology, Pune.

We also studied the related environmental factors such as the presence of mosquito nuisance and water collections in tanks, desert coolers, cattle sheds, broken vessels, pots, etc. Concurrently, using pamphlets printed in local Marathi language, we educated the local population regarding personal, family, and community protection against mosquito bites and mosquito source reduction.

Chikungunya infection is not new to India. Outbreaks have occurred in Calcutta(1) and south India,(2) with large numbers of people reporting febrile illness accompanied by the characteristic joint pains. In these outbreaks both sexes were equally affected. The chikungunya virus was first isolated in India in Calcutta in 1963[1] and in southern India (Vellore and Madras) in 1964[2]; 10 years later, in 1973, it was also identified in Solapur district of Maharashtra.[3]

In the present epidemic, also, the classical symptoms of fever with joint pains were observed. In the acute stage, the patient has difficulty in walking due to the pain and adopts a bent/flexed posture. The present outbreak has affected a large number of people as has been observed in previous outbreaks also.[4] The occurrence of multiple disease cases in some families over a short period of time indicates mechanical transmission of the disease. Some serological studies conducted in Calcutta[5] and Madras[6] have suggested that waning herd immunity, with accumulation of a susceptible population, could be one of the reasons for the occurrence of large epidemics at intervals of several.

The usual incubation period of chikungunya fever is 2–4 days. The illness is self-limiting. The acute symptoms include fever, headache, arthralgia, nausea, vomiting, abdominal pain, rash, and malaise.

Diagnosis is on the basis of symptoms and by detection of antigens or antibodies, molecular techniques like polymerase chain reaction (PCR), and virus isolation by cell culture. Treatment is supportive/palliative, with complete bed rest and simple analgesics like paracetamol, ibuprofen, etc. Aspirin should be avoided as it may cause Reye's syndrome.

The preventive measures at the individual level include use of mosquito repellents like coils, mats, body creams, mosquito nets, etc. At the community level it is important to ensure that there are no collections of water in household vessels or around dwelling places. The usual recommendation is that on every fifth day all vessels containing water should be emptied; this observance of a ‘dry day’ breaks the life cycle of the mosquito.

The outbreak of chikungunya fever may be due to a variety of social, environmental, behavioral, and biological changes. Lack of use of personal protective measures, ignorance, and poor environmental conditions seem to be responsible for the epidemic.
